# AlphaGenome: a framework for integrated regulatory variant interpretation

**DOI:** 10.7150/ijbs.133555

**Published:** 2026-03-30

**Authors:** Taeho Kwon, Hakjin Kim, Sun-Uk Kim, Seon-Kyu Kim

**Affiliations:** 1 Futuristic Animal Resource and Research Center, Korea Research Institute of Bioscience and Biotechnology (KRIBB), Cheongju, Chungbuk 28116, Republic of Korea.; 2 AI-Bio Solution Team, Genomic Medicine Research Center, Korea Research Institute of Bioscience and Biotechnology (KRIBB), Daejeon 34141, Republic of Korea.; 3 Advanced Bioconvergence Department, Department of Bioscience, Department of Bioinformatics, KRIBB School, Korea National University of Science and Technology (UST), Daejeon 34113, Republic of Korea.; 4 Quantum AI Bio Research Laboratory (KJQI-JQL), In Quantio, Gene on Biotech, Daejeon 35229, Republic of Korea.

In a recent study published in *Nature*, Avsec *et al.* describe AlphaGenome, a deep learning model designed to predict the effects of genetic variants on regulatory genomic features through the integration of long-range DNA sequence context with base-pair-resolution, multimodal outputs 1. AlphaGenome simultaneously models transcription, splicing, chromatin accessibility, transcription factor binding, and three-dimensional chromatin interactions directly from primary DNA sequence 1. The study addresses previously reported trade-offs in sequence-to-function modeling and presents a unified approach for regulatory variant effect prediction.

Interpreting the functional consequences of non-coding genetic variants remains a major challenge in genomics 2. Although large-scale functional genomics efforts have generated extensive maps of regulatory elements across diverse cell types and tissues, translating sequence variation into mechanistic understanding remains difficult 2. Non-coding variants can influence gene regulation through multiple pathways, including altered transcription initiation, changes in RNA processing, modulation of chromatin accessibility, and perturbation of higher-order chromatin structure. These regulatory effects are frequently context dependent and may only become apparent through coordinated changes across several regulatory layers, which complicates both experimental characterization and computational interpretation 2.

Existing deep learning models for sequence-to-function prediction have achieved substantial progress, yet they remain constrained by architectural and computational trade-offs. Models optimized for nucleotide-level resolution typically operate on relatively short input sequences, which limits their ability to capture distal regulatory interactions such as enhancer promoter interactions 3. In contrast, models designed to incorporate long-range genomic context often rely on coarser output resolutions that can obscure fine-scale regulatory features such as splice sites or transcription factor binding motifs 3. In addition, as noted in the original AlphaGenome study, predictive performance for regulatory interactions may gradually decrease with increasing genomic distance from the target gene. Furthermore, many existing approaches are optimized for individual regulatory modalities, limiting their ability to capture coordinated regulatory effects across multiple layers of genome regulation 4.

AlphaGenome is designed to address these limitations through a unified modeling framework that integrates long-range sequence context, base-pair-resolution predictions, and multimodal regulatory outputs 1. The model processes up to 1 Mb of DNA sequence and predicts thousands of genome-wide functional tracks spanning transcriptional, post-transcriptional, epigenomic, and chromatin conformation-related modalities 1. Its architecture combines convolutional components for local sequence feature learning with transformer-based layers that capture long-range dependencies, enabling simultaneous representation of proximal regulatory elements and distal genomic interactions. This design allows the model to preserve fine-scale regulatory detail while maintaining sensitivity to distal elements that act over extended genomic distances.

A two-stage training strategy further contributes to the practical utility of AlphaGenome. During pretraining, the model learns to predict experimental genomic signals across multiple modalities and biological contexts 1. This is followed by a distillation step that consolidates predictive capacity into a single model instance suitable for efficient variant effect prediction. This strategy reduces reliance on model ensembles and facilitates scalable application to large variant datasets, which is particularly relevant for population-scale sequencing studies.

The performance of AlphaGenome was evaluated using a comprehensive set of benchmarks assessing both genome track prediction and variant effect prediction 1. These evaluations span a broad range of regulatory readouts, including gene expression, splicing, chromatin accessibility, transcription factor binding, and chromatin contact maps. Across these tasks, AlphaGenome matched or exceeded the performance of existing sequence-based models on most benchmarks. Importantly, the evaluations were conducted using standardized datasets and metrics, allowing direct and transparent comparison with previously published approaches 1. Together, these results indicate that AlphaGenome achieves competitive performance across diverse regulatory modalities within a single unified framework.

Variant effect prediction represents a central application of the AlphaGenome framework. By comparing predictions generated from reference and alternative sequences, the model estimates how specific genetic variants alter regulatory genomic features. This approach enables quantitative assessment of variant-associated changes rather than binary classification of variant functionality 2. Such quantitative predictions are particularly relevant for non-coding variants, where regulatory effects may be subtle and distributed across multiple molecular processes.

Splicing-related predictions highlight the breadth of regulatory processes captured by AlphaGenome 1. The model predicts splice site classification, splice site usage, and splice junction activity at base-pair resolution using RNA sequencing-derived training targets. These outputs allow assessment of how genetic variants influence splicing decisions, including changes in splice site utilization and junction strength. While accurately capturing tissue-specific splicing effects remains challenging in some contexts, the reported results demonstrate that AlphaGenome captures core features of splicing regulation that are directly relevant for regulatory variant interpretation 4.

Beyond individual regulatory modalities, AlphaGenome enables joint analysis of variant effects across multiple layers of genome regulation. This integrative capability supports mechanistic interpretation by allowing predicted changes in transcription, chromatin accessibility, transcription factor binding, and chromatin interactions to be evaluated together 5. The authors illustrate this approach using regulatory variants near the TAL1 oncogene, where the model's predictions align with previously described regulatory mechanisms. This example demonstrates how coordinated multimodal predictions can support interpretation of disease-associated non-coding variants without reliance on separate modality-specific models.

The ability to analyze variant effects across regulatory layers also has implications for understanding how genetic variation propagates through regulatory networks. Rather than acting through isolated molecular events, many non-coding variants exert their effects through interconnected regulatory processes. A unified modeling framework such as AlphaGenome provides a means to examine these connections systematically, offering insight into how sequence variation influences regulatory architecture at multiple levels.

From a broader perspective, AlphaGenome provides a framework for systematic analysis of regulatory variant effects in the context of large-scale genomic studies. As whole-genome sequencing efforts continue to identify extensive non-coding variation, computational approaches capable of integrating multiple regulatory readouts from sequence alone will become increasingly important. AlphaGenome addresses key methodological limitations of existing sequence-to-function approaches through its unified treatment of long-range genomic context and base-pair-resolution prediction.

Despite these advances, several limitations warrant consideration. AlphaGenome was trained on broad collections of functional genomics data derived primarily from reference cell lines and healthy tissues, which may constrain its ability to capture disease-specific regulatory alterations. For example, somatic mutations that rewire enhancer-promoter interactions in cancer or context-dependent regulatory changes in neurodegenerative disorders may not be fully represented in the training data. The authors acknowledge that accurately capturing tissue-specific expression deviations and intermediate splicing efficiencies remains challenging. Furthermore, while the model demonstrates strong performance on population-level quantitative trait locus benchmarks, translating these predictions to individual patient variants in clinical settings will require additional validation. The availability of pretrained model weights and source code enables domain-specific fine-tuning, which may be necessary to achieve the specificity required for particular disease contexts or understudied cell types. Future work integrating patient-derived or disease-model datasets during training or adaptation may help bridge the gap between general regulatory prediction and disease-specific variant interpretation.

In summary, AlphaGenome provides a unified sequence-based framework for predicting regulatory variant effects across multiple genomic modalities. The integration of long-range genomic context with base-pair-resolution predictions addresses key limitations of existing sequence-to-function methods. As large-scale sequencing studies continue to identify extensive non-coding variation, models such as AlphaGenome may support systematic analysis of regulatory variant effects in both basic research and clinical variant interpretation. Taken together, continued advances in model architectures and training strategies, together with the integration of additional experimental modalities and species-specific data, may further enhance the utility of unified sequence-to-function frameworks.

## Figures and Tables

**Figure 1:**
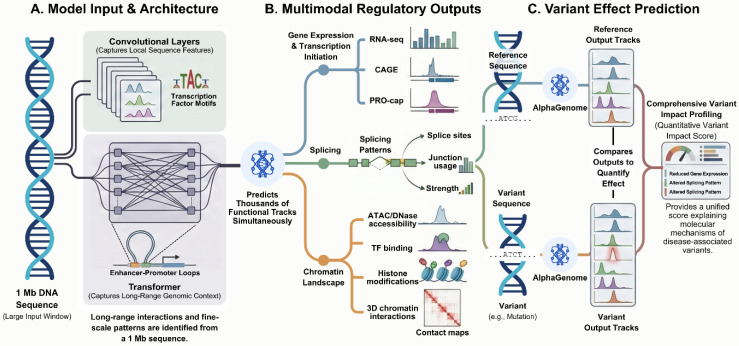
** Integrated sequence-based framework for regulatory variant interpretation by AlphaGenome.** (**A**) AlphaGenome takes a genomic DNA sequence of up to 1 Mb as input, which is processed by convolutional layers capturing local sequence features and transformer-based components modeling long-range genomic interactions. (**B**) The model outputs regulatory genomic features at base-pair resolution across multiple modalities, including transcription, splicing, chromatin accessibility, transcription factor binding, and chromatin interactions. (**C**) Variant effects are quantified by comparing regulatory output tracks predicted from reference and variant sequences, enabling quantitative assessment of regulatory changes associated with genetic variants. Figure created with TOV studio (https://www.tov.studio/).
